# Negative frequency-dependent selection is intensified at higher population densities in protist populations

**DOI:** 10.1098/rsbl.2015.0192

**Published:** 2015-06

**Authors:** Ewan J. A. Minter, Phillip C. Watts, Chris D. Lowe, Michael A. Brockhurst

**Affiliations:** 1 Department of Biology, University of York, Wentworth Way, York, Yorkshire YO10 5DD, UK; 2 Department of Ecology, University of Oulu, PO Box 3000, 90014 Oulu, Finland; 3 Centre for Ecology and Conservation, College of Life and Environmental Sciences, University of Exeter, Cornwall Campus, Falmouth TR10 9FE, UK

**Keywords:** selection, frequency dependence, density dependence, diversity, plankton

## Abstract

Natural populations of free-living protists often exhibit high-levels of intraspecific diversity, yet this is puzzling as classic evolutionary theory predicts dominance by genotypes with high fitness, particularly in large populations where selection is efficient. Here, we test whether negative frequency-dependent selection (NFDS) plays a role in the maintenance of diversity in the marine flagellate *Oxyrrhis marina* using competition experiments between multiple pairs of strains. We observed strain-specific responses to frequency and density, but an overall signature of NFDS that was intensified at higher population densities. Because our strains were not selected *a priori* on the basis of particular traits expected to exhibit NFDS, these data represent a relatively unbiased estimate of the role for NFDS in maintaining diversity in protist populations. These findings could help to explain how bloom-forming plankton, which periodically achieve exceptionally high population densities, maintain substantial intraspecific diversity.

## 1. Introduction

Many free-living protists exhibit high levels of intraspecific diversity [1–4] despite their large population sizes, which offer the potential for natural selection to operate efficiently and fix the fittest genotype(s). Negative frequency-dependent selection (NFDS) is a general mechanism that can maintain intraspecific diversity [5–7]. NFDS favours rare genotypes which subsequently increase in frequency to become common and are therefore disfavoured by selection, thereby allowing multiple genotypes to stably coexist.

NFDS is likely to interact with population density [8–14], becoming stronger at higher population densities owing to the intensification of competition, which could increase the potential for NFDS to maintain intraspecific variation. This interaction between NFDS and population density was first experimentally observed more than half a century ago in classic experiments with insects [8,9] and has since been examined theoretically [10,11] and observed empirically in a range of species [12–14]. Largely owing to the difficulties in distinguishing among multiple genotypes of protist species there have been no experimental tests of a role for NFDS in the maintenance of genetic diversity in these ecologically important organisms. The recent development of molecular methods now means it is feasible to study frequency-dependent intraspecific competition in protists [15].

Here, we provide, to our knowledge, the first experimental test for the operation of NFDS in a protist, the model flagellate *Oxyrrhis marina*. We estimated selection coefficients [16] for multiple pairs of strains at a range of starting frequencies and at several population densities that were representative of natural populations [17]. We observed strain-specific variation in responses but an overall signature of NFDS which was strengthened at higher population densities, suggesting a role for NFDS in maintaining the high intraspecific diversity observed in many natural protist populations.

## 2. Methods and materials

### (a) Model species

We quantified instantaneous selection rates [16] using seven strains of the marine flagellate *O. marina* Dujardin 1895 that were isolated from European North Atlantic coastal sites: EST02 (Estoril, Portugal), FAR01 (Faro, Portugal), ROS03 (Roscoff, France), PLY01 (Plymouth, UK), BGN01 (Bergen, Norway), BOD01 (Bodø, Norway) and TMO01 (Tromsø, Norway; electronic supplementary material, table S1). All strains of *O. marina* were isolated from seawater samples taken from tide pools and maintained at 16°C at a light intensity of approximately 80 μmol photons m^−2^ on a 14 L : 10 D cycle [18]. Media was 32 PSU sterile filtered artificial seawater (SASW) enriched with *f*/2 (Sigma Aldrich, UK) and inoculated with *Dunaliella primolecta* at a cell density of approximately 3 × 10^5^ cells ml^−1^ as a prey. Stock cultures were sub-cultured once per month. Pre-experimental cultures were created at least one month prior to experiments, without addition of *f*/2, and by replacing *D. primolecta* with heat-killed *Escherichia coli* [18] at a density of 1.25–2.5 × 10^6^ CFU ml^−1^ as food. Depending on *Oxyrrhis* density, fresh food was added to cultures every 2–5 days.

### (b) Frequency-density effects on selection experiments

Selection experiments to test for frequency and density dependence were performed by co-culturing six pairs of strains to estimate instantaneous selection coefficients [16]. Strain pair combinations were selected on the basis of the strain pairs differing at microsatellites alleles so that we could use microsatellite genotyping assays to estimate strain frequencies [15]; choice of pairs is therefore random with respect to strain ecological characteristics. Experimental microcosms were initiated at three initial frequency treatments (0.1, 0.5, 0.9) of the target strain and three total population density treatments (500, 2000 and 5000 cells ml^−1^) in a full factorial design with three replicates for each combination. Microcosms were 50 ml centrifuge tubes containing 50 ml SASW and heat-killed *E. coli* at a density of approximately 1.25–2.5 × 10^6^ CFU ml^−1^. After gentle mixing, 10 ml subsamples were taken from each microcosm at 0 and 48 h and the frequency of each strain was estimated using allele specific quantitative-PCR on microsatellite loci [15] (electronic supplementary material, Methods S1). Given a growth rate of approximately 0.388 ± 0.05 d^−1^ in our *O. marina* strains, the short incubations prevented the realized population densities of density treatments from overlapping even with exponential growth.

### (c) Data analysis

Selection coefficients (*s*), a measure of the rate of change in strain frequencies, for a target strain versus a non-target strain were estimated from the slope of the natural log of the strain ratio versus time [16]. A global model (i.e. including all pairwise assays) of selection coefficients was analysed using a mixed effects model, with random slopes and *s* as the dependent variable, density and frequency as fixed effects, and strain pair as a random effect using the R package ‘lme4’ [19]. Owing to the non-independent and reciprocal nature of selection coefficients (where for a given pair of strains the value of *s* for the target strain is equal to the negative value of *s* for its competitor) the strain with the positive mean *s* across treatments was designated the target strain.

To investigate interactions between density and frequency upon the strength of selection, further analyses were performed independently on frequency dependence within density treatments, by ANOVA, using a simple main effects test with an adjusted *α*-value of 0.017. Frequency and density dependence on selection coefficients was also tested for each pair of strains independently by two-way ANOVA, with *s* as the dependent variable and frequency and density as factors. Owing to the arbitrary designation of a strain as ‘target’ (i.e. based on it having a mean positive selection coefficient) and to test for competitor-specific effects against the same strain, we performed additional analyses by assigning two ‘focal’ strains: EST02 (Portugal) and BGN01 (Norway) which represent different populations of origin.

All statistical analyses were conducted in R v. 3.1.0 (R Core Development Team, 2014) and all data are presented as mean ±1 s.e.

## 3. Results

### (a) Frequency- and density-dependent selection

**Figure 1.**
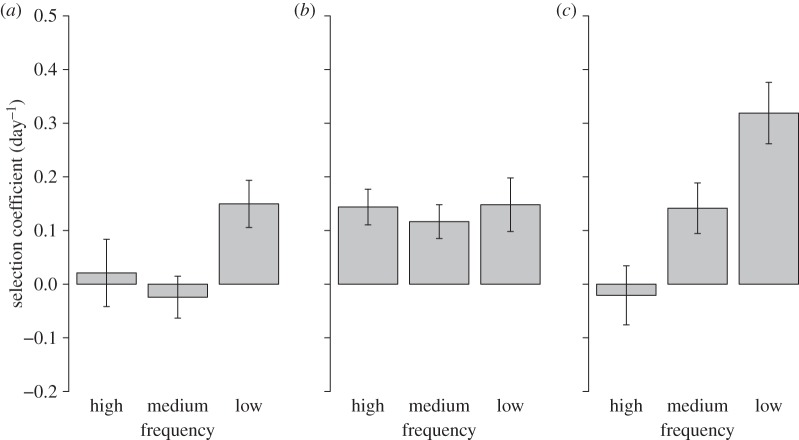
Mean selection coefficient for seven strains of *Oxyrrhis marina* relative to a competitor at (*a*) low (500), (*b*) medium (2000), and (*c*) high (5000 cells ml^−1^) population densities (panels) and high (0.9), medium (0.5) and low (0.1) initial frequencies (bars).

Across all experiments there were significant interactions between frequency and density, frequency and strain pair, and density and strain pair on selection coefficients (mixed effects model, *χ*^2^_4_ = 26.7, *p* < 0.001). The significant interaction between frequency and density is explained by weak or a lack of significant frequency dependence at low (simple main effects ANOVA, *F*_2,51_ = 3.30, *p* = 0.048) and medium (simple main effects ANOVA, *F*_2,51_ = 0.19, *p* = 0.83) population densities but strong, significant NFDS at high population densities (simple main effects ANOVA, *F*_2,51_ = 10.16, *p* < 0.001; figure 1).

With EST02 and BGN01 as ‘focal’ strains, we observed similar responses of selection to frequency and density. For EST02, there was an interaction between frequency and density on selection (two-way ANOVA, *F*_4,70_ = 3.188, *p* < 0.05) that followed the pattern described above, but with no interaction with competitor strain; this suggests that EST02 responded to changes in frequency and density regardless of its competitor. For BGN01, there was a significant three-way interaction between density, frequency and the competitor (three-way ANOVA, *F*_8,54_ = 2.248, *p* < 0.05) that suggests a competitor-specific response to frequency and density for this strain.

Owing to the variation in responses exhibited by strain pairs, we also analysed the effects of frequency and density for each individual strain combination. Our experimental microcosms revealed complexity with all possible combinations of frequency dependence (BGN01 and TMO01), density dependence (ROS03 and EST02) and interactions between density and frequency dependence (PLY01 and BGN01; EST02 and BOD02), and two pairs of strains (EST02 and BGN01; EST02 and FAR01) showing no significant effect of frequency or density on selection (table 1). This suggests that the selection response to frequency and density also depends upon the combination of genotypes.

**Table 1.** Two-way ANOVA statistics for frequency and density dependence of selection coefficients in *Oxyrrhis marina* microcosms. (Model results are presented with interactions, where significant, or otherwise with both factors.)

target strain	competitor strain	factor	d.f.	sum squares	mean square	*f*	*p*-value
ROS03	EST02	density	2	0.3955	0.1978	8.984	0.0014
		frequency	2	0.1108	0.0554	2.517	0.1037
		residuals	22	0.4843	0.0220		
BGN01	TMO01	density	2	0.2115	0.1058	3.042	0.0682
		frequency	2	1.8189	0.9094	26.159	<0.0001
		residuals	22	0.7649	0.3048		
EST02	BOD02	density	2	0.0249	0.0124	0.697	0.5111
		frequency	2	0.0237	0.0119	0.664	0.5267
		density × frequency	4	0.2913	0.0728	4.080	0.0158
		residuals	18	0.3213	0.0179		
PLY01	BGN01	density	2	0.3337	0.1669	16.090	<0.0001
		frequency	2	0.1439	0.0720	6.940	0.0058
		density × frequency	4	0.2694	0.0673	6.494	0.0020
		residuals	18	0.1867	0.0104		
EST02	BGN01	density	2	0.0070	0.0035	0.161	0.852
		frequency	2	0.0612	0.0306	1.413	0.265
		residuals	22	0.4767	0.0216		
FAR01	EST02	density	2	0.0389	0.0194	0.767	0.476
		frequency	2	0.0677	0.0339	1.336	0.283
		residuals	22	0.5574	0.0253		

## 4. Discussion

Understanding the mechanisms that maintain intraspecific variation in natural populations is an important challenge in evolutionary ecology, as this variation underpins numerous fundamental processes [20,21], including adaptation to environmental change (e.g. [22]). We observed an overall signature of NFDS between pairs of competing strains of *O. marina*, which was intensified at higher population densities. This finding may be especially relevant to understand the evolutionary ecology of bloom-forming plankton, which periodically achieve exceptionally high population densities and yet often maintain substantial intraspecific diversity [23,24]. It is important to note, though, that within this overall pattern there was extensive strain-specific variation in the response to both frequency and density, highlighting the potential complexity of competitive interactions within natural protist populations. Nevertheless, because our strain selection was not based *a priori* on particular traits or phenotypes expected to exhibit NFDS (e.g. public goods scenarios in bacteria [25] or sexual selection between phenotypic morphs in animals [26]) our data represent a relatively unbiased estimate of the role of NFDS. Our data provide the first evidence to our knowledge that NFDS is a plausible mechanism maintaining the high levels of intraspecific diversity typical of natural free-living protist populations. The next challenge is to incorporate the interaction between frequency, density and genetic diversity into models that attempt to predict population dynamics in natural systems, for example, the seasonal dynamics of plankton blooms and the responses of protist populations to environmental change.

## Supplementary Material

Supplementary methods

## Data accessibility

Data available from the Dryad Digital Respository: http://dx.doi.org/10.5061/dryad.nk52s.
