# Supplementary material for: Negative frequency-dependent selection is intensified at higher population densities in protist populations
Source: Biol Lett. 2015 Jun;11(6):20150192. doi: 10.1098/rsbl.2015.0192 (PMC4528467; doi:10.1098/rsbl.2015.0192)
Supplement: Supplementary methods [file rsbl20150192supp1.docx]

**Supplementary Information (SI)**

**SI Methods** (Modified from Minter et al. (2015)

AsQ-PCR calibrations were performed using DNA extracted with Chelex-100™ (Walsh *et al.*, 1991), a fast and inexpensive DNA extraction method. Here, the target DNA was a series of mixed cultures to simulate a protist microcosm experiment: *O. marina* cultures were diluted to 1,500 cells ml^-1^ for the strain pairs used in this study then mixed at four ratios of 1:24, 1:4, 4:1 and 24:1 to a total volume of 50 mL. Samples were centrifuged and genomic DNA extracted from the cell pellet by adding 50 μL Chelex-100™ (Bio-Rad Laboratories) solution (5% w/v) and then incubating the samples at 95°C for 1 hour. PCR amplification of one of four microsatellite loci (MS_23, MS_30, MS_37 and MS_41) was performed for each sample (and in replicate *n*=8) in 10 μl reaction volumes that contained Green GoTaq® reaction buffer (pH 8.5) (Promega), 200 μM each dNTP, 1.5 mM MgCl_2_, 10 μg BSA, 0.25 units GoTaq® DNA polymerase (Promega), 0.3 pmol of forward primer that was 5’-labelled with either 6-fam, ned, pet or vic flourophores (Applied Biosystems), 0.3 pmol reverse primer, and with 2 μL of the Chelex-100^TM^-extracted DNA as a template DNA. Thermal cycling conditions were: 95°C for 1 min, N_c_×[95°C 30 s, 58°C 45 s, 72°C 45 s], 72°C 10 min, where N_c_ represents the number of cycles (N_c_= 33, 32, 29, & 32 for MS_23, MS_30, MS_37, & MS_41 respectively). PCR products were pooled with Genescan 500 liz size standard (Applied Biosystems) and separated by electrophoresis on an AB3130xl (Applied Biosystems). Expected ratios of the pair of strains were calculated from the mix ratio and a triplicate cell density count of each population, performed using a Sedgewick Rafter Chamber. Allele sizes and peak heights were determined using Genemapper v.3.0 (Applied Biosystems). These data were then transformed to a peak height ratio (PHR) - the ratio between the peak heights of the alleles generated by the two strains - which was natural log-transformed prior to analysis in all cases. Calibration curves were created using linear least-squares regression (Levenberg-Marquardt Algorithm). AsQ-PCR on experimental samples was performed as above (n=4 for each sample). Strain frequencies were back calculated using the strain-pair specific calibration curve.

**SI Table 1.** Information about the strains of *Oxyrrhis marina* used in this study.

| Strain ID | Sampling Location | Latitude | Longitude | Sampling Date |
| --- | --- | --- | --- | --- |
| TMO01 | Tromso, Norway | 69° 37' 48" | 18° 54' 36" | 15/08/2011 |
| BOD02 | Bodo, Norway | 67° 16' 33" | 14° 34' 14" | 25/06/2011 |
| BGN01 | Bergen, Norway | 60° 14'8" | 5° 11'8" | 22/07/2009 |
| PLY01 | Plymouth, UK | 50° 21' 48" | -4° 8' 21" | 25/04/2008 |
| ROS03 | Roscoff, France | 48° 43' 39" | -3° 59' 22" | 17/05/2009 |
| EST02 | Estoril, Portugal | 38° 42' 7" | -9° 23' 35" | 09/07/2009 |
| FAR01 | Faro, Portugal | 37° 1' 1" | -7° 59' 36" | 21/01/2008 |

**SI References**

Walsh, P., Metzger, D., & Higuchi, R. (1991) Chelex 100 as a medium for simple extraction of DNA for PCR-based typing from forensic material. *BioTechniques*, **10**, 506-513.
